# Eradication success for non-tuberculous mycobacteria in children with cystic fibrosis

**DOI:** 10.1183/13993003.03636-2020

**Published:** 2021-05-27

**Authors:** Dominic A. Hughes, Idan Bokobza, Siobhán B. Carr

**Affiliations:** 1 National Heart and Lung Institute, Imperial College London, London, UK; 2 Royal Brompton and Harefield NHS Foundation Trust, London, UK

## Abstract

Non-tuberculous mycobacteria (NTM) are an emerging pathogen worldwide in both cystic fibrosis (CF) and non-CF pulmonary disease (PD), with reports suggesting an increasing prevalence [1, 2]. It is an opportunistic infection acquired from the environment [3], though conflicting evidence remains around person-to-person transmission [4, 5]. Recent evidence suggests that *Mycobacterium abscessus* complex (MABSC) may be the most detrimental airway infection to lung function in CF [6], yet its treatment remains poorly evidenced.

## Abstract 2

**Non-tuberculous mycobacteria pulmonary disease affects cystic fibrosis lung health and increasingly affects children. This study reports treatment outcomes from a large CF centre, with eradication rates exceeding 60% for *M. abscessus* complex infection.** https://bit.ly/2L8TdFM

*To the Editor*:

Non-tuberculous mycobacteria (NTM) are an emerging pathogen worldwide in both cystic fibrosis (CF) and non-CF pulmonary disease (PD), with reports suggesting an increasing prevalence [1, 2]. It is an opportunistic infection acquired from the environment [3], though conflicting evidence remains around person-to-person transmission [4, 5]. Recent evidence suggests that *Mycobacterium abscessus* complex (MABSC) may be the most detrimental airway infection to lung function in CF [6], yet its treatment remains poorly evidenced.

The paediatric CF service at the Royal Brompton Hospital (RBH) has had standardised diagnostic and treatment guidelines since 2011, which follow the American Thoracic Society (ATS) guidelines for the diagnosis of NTM-PD [7] and latterly CF-specific consensus guidelines [8, 9]. In CF, the two most commonly encountered species are MABSC and *Mycobacterium avium* complex (MAC), with the former more prevalent in the UK and the latter in the USA [1, 10]. A recent consensus statement on treatment outcome definitions could not reach consensus on a definition of cure, but a microbiological cure was agreed as the end of anti-microbial treatment after culture conversion [11].

There are no published paediatric or adult CF randomised controlled trials of NTM treatment. Additionally, there are no established estimates of eradication success for NTM-PD in children, while there are limited reports from mixed adult/paediatric cohorts [12, 13]. This cohort study aimed to establish NTM prevalence and treatment success in children with CF, and to identify any factors that may affect treatment success.

Electronic records for all children at the RBH CF centre were reviewed to identify cases with at least one new respiratory NTM infection between 2011 and 2018. Samples for NTM were taken at annual review, opportunistically if productive of sputum and from bronchoalveolar lavage (BAL), then grown in liquid culture medium, speciated with the Hain NTM-DR Test, inducible macrolide resistance detected by the Hain assay and VNTR typing performed by a national reference laboratory. Demographic data, spirometry, treatment details and associated diagnoses were taken from the visit closest to the first NTM growth. Treatment decisions were based on local guidelines [14], also taking into account antimicrobial susceptibility testing. Airway samples were tested every 3 months throughout treatment and for 12 months following treatment completion. The time points captured were time to start of treatment from first growth, time to culture conversion, length of treatment, and successful eradication. Transient infection is any isolate that did not persist on subsequent (two or more) cultures despite no treatment. For our centre, “cleared” is considered as the date treatment was stopped after 12 months of negative cultures (microbiological cure [13]), and “eradicated” as 12 months of negative cultures following stopping treatment. Approval for the study was granted through the hospital audit office (ref 288700). IBM SPSS Statistics (version 24) was used. Statistical significance was assumed at p<0.05.

567 children with CF received care at RBH between 2011 and 2018. 63 new NTM isolates were found from 59 children (period prevalence 10.4%); 34 MABSC (28 *M. abscessus abscessus*, six *M. abscessus massiliense*), 21 MAC, and eight other NTM species (four *M. kansasii*, three *M. chelonae* and one *M. malmoense*, all of which cleared without treatment). A median of six samples were taken per patient during their treatment period. There was no difference in age, sex or body mass index z-score between groups, and all demonstrated highest prevalence in females (table 1). Median percent predicted forced expiratory volume in 1 s (ppFEV_1_) for those with MAC was lower in those who were treated, though this did not meet significance. All treated patients met ATS/Infectious Diseases Society of America criteria for NTM-PD [7].

**TABLE 1:** Demographics and outcomes of *M. abscessus* complex (MABSC), *M. avium* complex (MAC) and other non-tuberculous mycobacteria (NTM) groups

	**MABSC**	**MAC**	**Other NTM**
**Patients n**	34	21	8
**Sample type for first growth**			
Sputum	27 (79)	13 (62)	8 (100)
BAL	7 (21)^#^	8 (38)^¶^	0
**Age years**	11.8 (3.2–17.3)	12.7 (3.6–16.7)	11.6 (7.4–15.9)
**Female**	21 (62)	13 (62)	6 (75)
**BMI z-score**	−0.13 (−2.08–1.46)	−0.38 (−1.89–0.82)	0.45 (−1.01–1.28)
**ppFEV_1_**	80 (50–113)	80 (36–121)	95 (79–119)
**Transient infection**	7 (21)	11 (52)	8 (100)
**Commenced antibiotic treatment**	26 (76)	9 (43)	0
**Microbiology: PsA/Sa/Asp**	12 (46)/13 (50)/14 (54)	2 (22)/2 (22)/7 (78)	
**ppFEV_1_ starting treatment**	80 (50–113)	75 (52–89)	
**Time to treatment from first growth** **days**	102 (16–587)	518 (42–903)	
**Intravenous treatment initiation**	26 (100)	4 (44)	
2** **week duration	9 (35)	3 (75)	
3** **week duration	17 (65)	1 (25)	
**Clearance of NTM with treatment**	21 (81)	8 (89)	
**Eradication of NTM with treatment**	17 (65)	6 (67)	
**Failure of NTM treatment**	7 (27)	3 (33)	
**Duration of successful treatment** **months**	19 (4–33)	16 (12–22)	
**Duration of unsuccessful treatment** **months**	29 (15–48)	20 (18–28)	

Data are presented as n (%) or median (range), unless otherwise indicated. ^#^: five had confirmation on subsequent sputum samples; the two patients diagnosed on bronchoalveolar lavage (BAL) alone had repeat BAL to look for clearance. ^¶^: four had confirmation on sputum and went on to treatment; two had repeat negative BAL samples, two negative subsequent sputum and were not treated. BMI: body mass index; ppFEV_1_: percent of predicted forced expiratory volume in 1 s; PsA: *Pseudomonas aeruginosa*; Sa: *Staphylococcus aureus*; Asp: *Aspergillus* sp.

For patients isolating MAC, 11 had transient infection (52%), nine commenced treatment (43%) and one did not start treatment. Four received intravenous (*i.v.*) initiation treatment with amikacin and meropenem. All treated patients received triple oral antibiotic maintenance therapy that included ethambutol and a macrolide. Six of the nine treated eradicated the infection (67%), three of which had received *i.v.* initiation therapy. The most frequently seen co-infection within 1 year of starting treatment was *Aspergillus fumigatus*, found in seven (78%).

Of those that grew MABSC, 26 started treatment (76%), seven had transient infection (21%) and one did not start treatment. All received *i.v.* initiation antibiotics, the majority for 3 weeks. All had triple antibiotic *i.v.* initiation regimes (all had amikacin, 24 meropenem with either cefoxitin or tigecycline) and a daily oral macrolide, regardless of *erm-41* status. For the maintenance phase, all patients received nebulised amikacin in combination with dual or triple oral antibiotics including a macrolide. Of those treated for MABSC-PD, 21/26 (81%) initially “cleared” the infection and 2/21 (10%) of these regrew after stopping treatment. Overall, 17/26 (65%) achieved eradication, 7/26 (27%) failed to eradicate, and 2/26 (8%) cleared the infection but transitioned before 12-month post-treatment follow-up. One of those who eradicated withdrew from full treatment after 4 months (poor drug tolerance). Eradication rates for *M. abscessus massiliense* were higher overall: 4/5 (80%) eradicated with treatment and one spontaneously cleared. Receiving 2 weeks compared to 3 weeks of initiation treatment had no effect on eradication rates (63% *versus* 69%; nonsignificant). ppFEV_1_ at diagnosis did not predict eradication success (mean 81% pred *versus* 82% pred; nonsignificant). A functional *erm*-*41* macrolide-resistance gene was identified in 10/16 (63%) of *M. abscessus abscessus* first isolates that were tested and treated. Of the 13/16 that had completed treatment and 12-month post-treatment observation: 5/8 (63%) eradicated if *erm*-*41* active *versus* 4/5 (80%) when *erm*-*41* inactive. As with MAC patients, the most frequently occurring co-infection within 12 months of NTM isolation was *Aspergillus fumigatus* (54%). Five of the MABSC group grew more than one species of NTM during the study period.

A sputum sample was successfully obtained in 23 (92%) children at 6 months of treatment for MABSC-PD. In those going on to eradicate, 82% (14/17) had achieved culture conversion by then, compared to 29% (2/7) who failed to eradicate. Within our study period, we saw one regrowth 12 months after clearance that later cleared without treatment. ppFEV_1_ recorded at the start and end of treatment (taken 20 months after starting treatment for those who failed eradication) showed a 7% improvement for those who successfully eradicated or cleared MABSC, in contrast to a 3% decline in those who failed treatment.

This study from a large single centre aimed to establish NTM treatment outcomes in children with CF. This is especially pertinent in an era of increasing prevalence, with growing evidence of impact on outcomes and lung transplant eligibility [15].

The overall period prevalence of 10.4% is in line with current rates in the UK. Our data show higher transient infection rates for MAC and other NTM species compared to MABSC, which may reflect a less aggressive treatment start strategy for non-MABSC infections. This may also explain why patients treated for MAC-PD trend towards a lower ppFEV_1_ compared to those not treated. As expected, the duration of treatment is longer in those who fail to eradicate: the decision to cease treatment in patients with persistent positive cultures remains difficult.

Our data demonstrate an overall eradication rate of 65–73% for MABSC-PD (depending on whether two patients lost to follow-up subsequently “eradicated”), and 78% for MAC-PD. These rates are significantly higher than the 20–40% previously reported [12, 13], which may reflect lower levels of underlying lung disease in a paediatric population. This information will be valuable when discussing NTM treatment with patients and families. Early identification and treatment for MABSC in particular is thought to positively impact on eradication rates, placing additional emphasis on screening policies such as the use of induced sputum in non-productive patients [16].

These data have already affected practice within our centre; culture positivity after 6 months of treatment for MABSC-PD is seen as predicting treatment failure and a second course of *i.v.* antibiotics is given in the hope this will improve eradication rates. Our relatively small sample size makes it challenging to identify beneficial outcomes from eradication, though indications from ppFEV_1_ at the start and end of treatment suggest that eradication of MABSC is beneficial to lung health.

This is the first study to report NTM treatment outcomes specific to children with CF, and to suggest potential strategies for improving treatment success. A prospective randomised controlled trial is urgently needed.

## Shareable PDF

10.1183/13993003.03636-2020.Shareable1 This one-page PDF can be shared freely online.
Shareable PDF ERJ-03636-2020.Shareable
