# Supplementary material for: Eradication success for non-tuberculous mycobacteria in children with cystic fibrosis
Source: Eur Respir J. 2021 May 27;57(5):2003636. doi: 10.1183/13993003.03636-2020 (PMC8280568; doi:10.1183/13993003.03636-2020)
Supplement: Supplementary file 1 [file ERJ-03636-2020.Shareable.pdf]

SHAREABLE PDF

Eradication success for non-tuberculous mycobacteria in children with cystic fibrosis

Dominic A. Hughes[1,2], Idan Bokobza[2] and Siobhán B. Carr[1,2]

Affiliations: [1]National Heart and Lung Institute, Imperial College London, London, UK. [2]Royal Brompton and Harefield NHS Foundation Trust, London, UK.

Correspondence: Dominic A. Hughes, National Heart and Lung Institute, Emmanuel Kaye Building, 1B Manresa Road, London, SW3 6LR, UK. E-mail: d.hughes17@imperial.ac.uk

@ERSpublications
Non-tuberculous mycobacteria pulmonary disease affects cystic fibrosis lung health and increasingly affects children. This study reports treatment outcomes from a large CF centre, with eradication rates exceeding 60% for *M. abscessus* complex infection. https://bit.ly/2L8TdFM

Cite this article as: Hughes DA, Bokobza I, Carr SB. Eradication success for non-tuberculous mycobacteria in children with cystic fibrosis. *Eur Respir J* 2021; 57: 2003636 [https://doi.org/10.1183/13993003.03636-2020].

This single-page version can be shared freely online.

To the Editor:

Non-tuberculous mycobacteria (NTM) are an emerging pathogen worldwide in both cystic fibrosis (CF) and non-CF pulmonary disease (PD), with reports suggesting an increasing prevalence [1, 2]. It is an opportunistic infection acquired from the environment [3], though conflicting evidence remains around person-to-person transmission [4, 5]. Recent evidence suggests that *Mycobacterium abscessus* complex (MABSC) may be the most detrimental airway infection to lung function in CF [6], yet its treatment remains poorly evidenced.

Copyright ©The authors 2021.. This version is distributed under the terms of the Creative Commons Attribution Licence 4.0.
